# Framework proposal for Role-Playing Games as mental health intervention: the Critical Skills methodology

**DOI:** 10.3389/fpsyt.2024.1297332

**Published:** 2024-04-25

**Authors:** Victor Henrique Oyamada Otani, Rafael A. C. B. Novaes, Julia Pedron, Pedro Chen Nabhan, Thaísa Malbar Rodrigues, Ryo Chiba, João Vitor Cardoso Guedes, Lucas Murrins Marques, João Ricardo Nickenig Vissoci

**Affiliations:** ^1^ Mental Health Department, Santa Casa de Sao Paulo School of Medical Sciences, São Paulo, Brazil; ^2^ Psychobiology Department, Federal University of São Paulo (UNIFESP), São Paulo, Brazil; ^3^ Independent Researcher, São Paulo, Brazil; ^4^ Division of Translational Health Sciences, Department of Emergency Medicine, Duke Global Health Institute, Duke University, Durham, NC, United States

**Keywords:** gamification, social skills, cognitive behavioral therapy, Consolidated Framework for Implementation Research, Role-Playing Games (RPGs)

## Abstract

Gamified interventions are an emerging approach in mental health treatment and prevention. Their positive effects on managing various clinical conditions stem from enhancing social skills. However, cost-effective options like Table-top Role-Playing Games (TTRPGs), which offer similar benefits to other game-based interventions, lack standardized methods for ensuring replicability. In this regard, the method outlined in this study endeavors, in a structured and guided manner drawing from the Consolidated Framework for Implementation Research (CFIR), to establish a six-step protocol for developing an intervention method utilizing TTRPGs. In all Steps, we aim to anchor ourselves in robust literature concerning social skills training (SST), cognitive behavioral therapy (CBT), and gamification comprehensively. Thus, the method presented encompasses the objectives of SST, the strategies of CBT, and the dynamics of gamification via TTRPGs. Furthermore, we demonstrate a possible application of the method to illustrate its feasibility. Ultimately, the final method is structured, evidence-based, easily applicable, cost-effective, and thus viable. Mental health professionals seeking a structured and instructional tool for protocol development will find support in the method proposed here.

## Introduction

Mental health disorders represent a significant global challenge, being a leading cause of death and disability worldwide. They account for 7% of the global burden of disease and contribute to up to 19% of all years lived with disability (1).In the United States, around 2 out of 10 adults are affected by some form of mental illness, with approximately 4% experiencing a debilitating mental disorder (2).As the burden of mental health disorders continues to rise, the World Health Organization (WHO) emphasizes the necessity for preventative strategies due to the current constraints of scalable treatment modalities (3). This need is particularly urgent given the increasing burden in low- and middle-income countries, underscoring the importance of low-cost and task-shared interventions.

A promising approach to promoting mental health has been through social skills training (SSTs) (4). Social skills training integrated with cognitive behavioral therapy (CBT) has demonstrated effectiveness in improving depressive symptoms (5) and symptoms related to autism (6, 7).

Applied games interventions are an emerging modality in the field of mental health promotion. By utilizing games originally developed for entertainment or games with an interventional purpose (e.g., serious games), numerous initiatives have reported positive outcomes in psychological well-being, depressive or anxiety disorders (8, 9), social anxiety (10, 11), disruptive behaviors (12, 13), schizophrenia (14), ADHD (15), autism (16), or resilience enhancement (17).The rationale behind such interventions is that immersive, ludic, and challenging gamified environments can enhance treatment engagement, facilitate the learning of new behavioral repertoires, increase emotional awareness, and improve social skills (18–21). However, digital gamified solutions are often costly, difficult to adapt across different scenarios, population groups, or diseases (22). Additionally, video games may lead to user fatigue due to repetitive experiences and lack the versatility required to ensure a therapeutic environment (23, 24). One solution to these limitations is to leverage the gamified and experiential narrative structure of Table-top Role-Playing Games (TTRPGs).

TTRPGs are uniquely suited to be used as interventional tools. As cooperative group-based table-top games, one only needs a pen and paper to be able to play. TTRPG sessions typically involve a story, a narrative arc, and a series of challenging situations that encourage players to embody their game characters, make choices, and exhibit their characteristics. In these games, a storyteller (often referred to as the Game Master or Keeper, here termed “The Guide”) guides the players through the unfolding storyline and its scenarios. The character’s abilities, strengths and flaws are determined within a set of rules that dictate the likelihood of success in various tasks. This game is highly customizable, with pre-defined storylines but real-time challenges and reactions (25, 26). TTRPG sessions are inherently gamified, designed to foster motivation, immersion, engagement, and entertainment through features such as rewards, progression, and points systems.

The structure and narrative of TTRPGs can be readily adapted to address therapeutic needs. As *the guide* narrates the story and the players make decisions, interact with the scenario and act-out different experiences, new behavioral and social repertories could be trained (26–28). The Guide plays a central role in the dynamics of the game and holds a unique position to continually evaluate, mediate, and intervene with the players. Moreover, TTRPGs could serve as a low-cost intervention. A Guide with training in counseling techniques, such as cognitive-behavioral therapy (CBT), could conduct interventions for groups in any location, language, and on multiple occasions.

Studies employing TTRPGs in interventions have reported positive experiences of improvements in mental health or educational outcomes (26, 29). Recent research indicates that TTRPGs can even reduce social anxiety, enhance social skills (30), and boost confidence in real-life social situations (31). However, to date, studies in the realm of mental health have primarily utilized TTRPGs in their original form, with minimal adjustments in their application. There exists a gap in the literature regarding studies that have adapted the structure of TTRPGs to serve as interventions for promoting mental health and social skills learning. Therefore, our objective with this study is to delineate the use of TTRPGs as an interventional tool to address the mental health burden, referred to as the Critical Skills Methodology. We aim to accomplish this by integrating the intervention mapping framework (32, 33) with TTRPG and CBT techniques. Additionally, we seek to demonstrate the implementation of this methodology with a cohort of psychiatry residents.

## Materials and equipment

### Intervention Mapping framework

The present methodology article outlines the development of an intervention based on the Intervention Mapping framework (34), and uses the Consolidated Framework for Implementation Research (CFIR) (35), whose domains serve as indicators for a pre-implementation formative evaluation, ensuring the structured parameters of the study. We have devised six structured steps for the development of an intervention proposal based on TTRPGs. In this context, in the following sections, we provide a detailed overview of the characteristics of each step. It is worth mentioning that this study has already received approval from the ethics committee at the *Santa Casa de Misericórdia de São Paulo* (CAAE: 40539920.0.0000.5479) and all participants provided their written consent prior to entering the program.

### Step 1. Defining the problem to be addressed through TTRPG

#### Targeted population, context, and key stakeholders

The initial step involved identifying the targeted population, contextualizing their environment, and recognizing the key personnel crucial to the success of the intervention. In our intervention framework proposal, we engaged with the population through an initial needs assessment to define their characteristics. This evaluation guided the development of the game narrative and determined the complexity of challenges or dilemmas introduced to facilitate the experiential learning component of the RPG session. Defining the population was essential for accurately identifying the mechanisms, scenarios, or flow of the session, and, most importantly, establishing the programmatic goals of the intervention.

In addition to identifying the targeted population, it is essential to understand the context in which the intervention takes place. Institutions possess unique cultures, norms, and standards that may potentially conflict with the intervention’s objectives. Moreover, the RPG session itself entails specific requirements and structures necessary for its implementation, as outlined in **Table 1**. These details may include the need for a private space for group sessions, minimal requirements for video conferences, discussions about rules and the complexity of the narrative, game mechanics, gamification strategies, and integration of sessions with the institution’s schedule, among others.

**Table 1:** Recommended requirements/structures for proper execution of the intervention in TTRPGs.

Elements	Context	Potential Pitfalls	Strengths
**Session lengths**	- RPG sessions can be lengthy, lasting several hours. For our planned intervention, we recommend sessions of 2 to 3 hours each. This duration is based on the average length of a Cognitive Behavioral Therapy (CBT) intervention, which typically spans 12 hours and is divided into 4 sessions.	- Some groups engage in non-stop play for 4 to 5 hours. While not a requirement, shorter sessions may lead to decreased engagement and intervention potential. - Long sessions could hinder the implementation due to participants’ potential inability to sustain engagement for such extended periods. - Long sessions might be tiring for new users. - Long sessions, particularly during extended combat sequences, may induce fatigue among certain players, particularly those experiencing issues related to inattention, hyperactivity, anxiety, or executive function deficits.	- Sessions of up to 3 hours allow all stages of the session script to be implemented satisfactorily, with time for rapport, updates, play and feedback. - This session time allows for increased player engagement and immersion in the narrative, providing ample opportunity for group discussions on decision making.
**Role Performance**	- In a TTRPGs, it is recommended (and encouraged) that players play their characters, with their voices, mannerisms and behaviors.	- More timid players may feel intimidated by this proposal, requiring greater flexibility and acceptance from the group, allowing them to feel free to act when, or if, they are ready.	- Role-playing enhances immersion in the game, as it allows players to connect more deeply with the narrative by embodying characters created within the proposed context. - In social skills training, role playing is essential as it enables individuals to practice and simulate behavioral rehearsals in problem situations. - Role-playing provides a valuable opportunity to rehearse thinking and acting from someone else’s perspective.
**Rules and Lore**	- Every game has a narrative set in a fantasy world, with problems and objectives elaborated according to the proposed “reality”. - In the project, we suggest a simplification of the elements of the game according to the group’s repertoire. It is more important for our objectives that the rules are adjusted to fit the players rather than vice versa. Additionally, we have included in the players’ planner methods for them to document their knowledge and orient themselves.	- For some players, a fantasy world with elements that are very far from our reality can cause confusion and disrupt the quality of immersion. It is important that players have enough repertoire to understand the proposed reality. - Very complex narratives, scenarios or objectives, without clear clues or references that allow players to orient themselves, can undermine player immersion and engagement.	- In a fictional world, anything is conceivable. Within this playful universe, situations and challenges can be crafted that are only feasible to experience in this manner. This offers richness to the experience, enabling the group to simulate challenges and suggest alternatives, experiencing the outcomes within the game without repercussions in the real world. - The operational rules aid in structuring the sessions so that players can engage with a well-organized world, where rights and responsibilities are equally distributed among all participants. - *The Guide* can reorganize the rules according to the group’s demand, allowing the expansion or restriction of behavioral alternatives, the simulation of norms and behaviors from other places (cultures) for players to train and the complexity (level of difficulty) of problem situations.
**Dice and Randomness**	- Dice are used to determine the success or failure of an action. In a THS, the focus is on the behavior presented and not the arbitrariness of luck. Therefore, it is recommended to adjust the challenge (minimum value to succeed in a dice roll) according to the quality of the player’s action. In situations where the player or group comes up with a very suitable solution*, The Guide* should consider whether or not the roll will be critical. In combat situations, the data must be kept, as it does not compromise training, adding strategy, flexibility and the need for group organization in an unpredictable context.	- Randomness Vs HS presented. Dice can antagonize the success of the player’s action.	- In combat, unpredictability drives the need for strategy. Players must pay attention to their teammates’ moves, predict and anticipate their actions, and remain flexible in response to the random results of the dice.
**Dynamics of battles**	- In some systems, battles are intrinsic to game mechanics. It is recommended that players have the ability to avoid them through behavioral rehearsal. They should be used as negative outcomes. However, some battles may be unavoidable, as the tactics of combat help in the organization, communication and strategy of the group.	- The battles are complex and require a lot of technical understanding of the characters’ abilities. Therefore, new players can be intimidated by this dynamic. - Some battles can last a long time, leading some players to become distracted and fatigued, causing them to lose attention and immersion in the game.	- Battles allow players to organize themselves and take turns, requiring constant communication (verbal and non-verbal) between group members. Players need to organize themselves strategically, highlighting points and compensating for each other’s weaknesses. - Battles require turn-taking, delaying rewards, observing, and paying attention to events to organize your actions. Therefore, they provide excellent training for planning, executing coordinated actions, monitoring, and group flexibility.
**Private location**	- TTRPGs take place in a safe, intimate, private and controlled environment.	Need for an isolated environment that provides privacy	- This safe space does not expose the group, allowing a more intimate bonding between the members and minimizing resistance/shyness in the dramatization of the characters.
**Number of sessions**	- TTRPGs do not have a session limit and can vary from games with just one encounter (called One Shots) or continuous tables, with longer narratives, reaching 10 or more sessions. - It is recommended that the number of sessions be defined after identifying the group’s needs. The more skills to train, the more sessions will be required. On average, one session is stipulated for each problem situation involving specific training in social skills. - We also recommend that *The Guide* create a One Shot to better understand the group, analyzing and identifying their in-game needs before determining the number of sessions.	- Storytelling over multiple sessions can lead to fatigue, resulting in the group becoming disoriented within the narrative and its recurring events. Therefore, it is crucial for *The Guide* to consistently assess the level of player engagement and interest. - At the same time, situations with few sessions can lead to a feeling of incompleteness. A journey must encompass a beginning, middle, and end, akin to a therapeutic session	- Not having a fixed number of sessions allows for greater flexibility in the intervention plan, as we acknowledge that each group possesses specific demands and needs. Just as a therapeutic plan varies according to the patient’s demands, the sessions also vary according to the training to be carried out, with real-time analysis of the group’s evolution and the goals achieved.
**Platform (Online vs Face-to-face)**	- TTRPGs can be played both in person and remotely. - Both modes are valid and offer unique gaming experiences, each with advantages and disadvantages. This adaptability allows training to be organized according to the demands (or possibilities) of group members.	- In the online modality, an internet connection, camera, microphone, and access to some communication tools are required. The need for these features may prevent some people from accessing training. - The face-to-face modality demands greater discipline from the group to meet at the place according to the agreed time and day. In large urban areas with restricted mobility, this can lead to delays, cancellations, and reduced engagement. Additionally, in epidemic scenarios like the current one, this mode of interaction poses health risks to participants.	- At the same time, in the online game, *The Guide* has more access and control over complementary media resources, such as music, ambient sounds, images, videos and much more. These features help players get visual and auditory information that can aid in their understanding and immersion world in the presented world. - At the same time, in person, players have better quality in communication and interaction. Here, not only can the quality of speech be observed, but body movements, expressions, and facial reactions in real time, which can aid in the interpretation of intentions, desires, and mental states of colleagues.
**Materials**	- In TTRPGs it is common to use complementary materials. We suggest: character sheet, dice (for successful or failed rolls in actions), sheets for notes (both for *The Guide* and for the players) and pencils/pens. In the online modality, this can be done using text files and virtual data scrolling. - Extra resources can be utilized, and we encourage their use. *The Guide* can incorporate music into the environment, utilize photos or images as references, create maps, and much more. These features are optional. - There are online platforms available that serve as virtual environments for playing RPGs, offering features such as character organization, scenery display, sound effects, and more. The use of these platforms is also optional.	- The amount of resources can directly impact the cost of sessions. - Excessive extra resources can distract from the main focus, diverting the group’s attention to stimuli that may be potentially irrelevant to the training.	- Although optional, the additional resources can greatly enhance the quality of immersion and motivation of the group during training. - Notes serve to help players organize their thoughts and actions. They are valuable not only as a record of events but also as a means of tracking plans, ideas, actions, consequences, potential alternatives, and goals. This aspect is crucial, as it enables players to analyze, reflect upon, and self-critique their training, a practice commonly employed in various psychological approaches.

Furthermore, each context involves stakeholders who play crucial roles in the intervention implementation. School teachers, section leaders, and mentors can offer valuable insights into defining program plans or understanding institutional culture. It is important to recognize that the intervention’s goals may vary depending on the perspectives of different stakeholders. For instance, the objective of the intervention may differ between the therapeutic perspective of an alcohol use clinic and the personal needs of individual participants. Mapping out the key stakeholders and their association with the targeted population is essential for developing the intervention and defining its feasibility.

#### Needs assessment

In designing an intervention based on TTRPGs for social skills training, a comprehensive needs assessment is essential to understand contextual issues, main determinants, and drivers of social skills issues in the given scenario (see **Table 2**). The needs assessment should encompass a range of mixed methodologies. Interviews and focus groups are ideal for comprehending the needs and designing interventions in specific areas of implementation. For our framework, which targets social skills, conducting thorough interviews on social skills repertory is recommended to establish baseline intervention goals. The use of validated measures is also advisable and can serve as important tools for demonstrating improvement over time and providing feedback to participants. However, the needs assessment should also involve key stakeholders, including parents and education professionals for interventions involving underage participants, healthcare professionals for interventions with clinical populations, and team leaders in organizational settings.

**Table 2:** Intervention planning logic model.

Targeted population	Stakeholders	Context	Needs assessment	Program goals	Program Team and sustainability
-Who is this intervention targeting?	-What are the social actors around the targeted population?	-Where is this intervention taking place?	-What is the outcome to be changed by the intervention?	-How will the intervention change the needs of this population?	-Who will deploy the intervention and ensure its continuation?
-How is this group going to be enrolled in the interventions?	-How is the environment engaged in the intervention?	-How will the intervention be deployed in the institution?	-How will the needs be assessed?	-How will the intervention change the targeted needs?	-How will the intervention be carried out?
-Barriers and facilitators -Implementation Climate -Knowledge and beliefs	-Relative Advantage -Barriers and facilitators -Implementation Climate -Readiness for implementation	-External policies and incentives -Complexity -Structural Characteristics -Culture -Readiness for implementation	-Targeted needs assessment Self-efficacy Stages of change	-Evidence strength and quality -Adaptability -Trialability -Complexity -Cost -Design Quality and Packaging -Planning	-Structural Implementation Climate -Readiness for implementation -Engagement

#### Program goals and processes

These assessments will inform the development of the intervention program by identifying goals (e.g., specific social skills to be trained) and the mechanisms to achieve these goals (e.g., RPG session structure, dynamic scenarios). Goals should be established based on a theoretical framework that guides intervention development. In the case of social skills training, the framework of social skills will inform goal setting based on the requirements of social skills met, mapped after RPG mechanistic characteristics. For example, if a team leader intends to use this intervention to promote empathy, the scenes and narrative should focus on creating dramatic opportunities to elicit empathetic situations for the participants to experience. The intervention method should consider these goals when designing the processes.

#### Intervention team and training

In a typical TTRPG session, there is a game master (referred to here as “The Guide”) and other players. Building on research from focus groups and psychodrama, we also propose the inclusion of an auxiliary therapist (referred to here as “Meta Guide”). The Meta Guide will be positioned among the players to assist The Guide in conducting the narrative.

### Step 2. TTRPGs and the determinants of behavioral and environmental outcomes

Similar to most experiential learning activities, RPGs can emulate situations requiring participants to utilize specific skills to achieve desired outcomes. However, RPGs possess inherent gamified characteristics that facilitate engagement, positive experiences, and learning (36).

#### Engagement characteristics

##### Adaptability

The intervention is built upon an open design framework, where *The Guide* is responsible for defining the objectives within the narrative. The model is designed to be dynamically updated based on participants’ progress. Scenes within the narrative are adaptable and lack predetermined outcomes, enabling *The Guide* to target various skills in each interaction. Rewards are allocated only to behaviors aligned with the intervention objectives.

##### Continuous Evaluation

Throughout the intervention, all participants will undergo assessment via online forms and rapport sessions. This approach allows *The Guide* to update intervention goals based on the most recent assessments submitted by the participants.

##### Gamification

Given the inherently playful nature of the game, it is anticipated that the incorporation of gamification dynamics will enhance motivation and engagement (37). *The Guide* will have the flexibility to enhance the fantasy and playful elements of the activity as needed to further boost engagement.

##### Role-Playing

Participants, when assuming roles within the game, are likely to experience a heightened sense of immersion (38). This immersion can be further intensified by *The Guide* addressing participants by their character names and incorporating each player’s preferences into their chosen role in the game.

##### Congruence

The intervention features a coherent narrative that incorporates elements of reality and facts alongside its fanciful aspects. This approach is designed to enhance participant immersion, fostering mindful engagement in the activity and facilitating emotional/cognitive connections with both the narrative and characters while minimizing feelings of estrangement. *The Guide* plays a pivotal role in maintaining this coherence throughout the intervention.

#### Intervention model

The intervention model was structured using the social skills training method, organized in the Theoretical-Practical Manual by (39). According to the authors, social skill is a descriptive construct and refers to behaviors that are appreciated by a culture, with positive outcomes for both the individual and their community (40–42). A Social Skill Training with RPG is a symbolic experience, which allows simulations of social situations in a fantasy world by using a playful/ludic context, allowing direct (and continuous) observation and evaluation of the players’ interaction and decision. In addition, complementary techniques and activities for social skills programs were adapted to the game’s context and implemented in the participants’ self-analysis and self-monitoring tasks (39).

To help *the Guide* observation and organize the players’ training process, cognitive behavioral techniques were included in the project. This approach presents a model that allows players to think about their thoughts in a structured way, bringing a clear distinction between events, thoughts, and feelings (29). To help players to analyze their thoughts, feelings, and behaviors, suggesting alternatives to their actions, two techniques were adapted and included in the project: the ABC (Activating - Beliefs - Consequences) technique and the construction of alternatives technique (43). Furthermore, the structure of a cognitive behavioral therapy session was adapted and used as a script for the RPG sessions, with Rapport (discussion about previous sessions), review of homework, updates, session summary and feedback (29).

#### Elements of the Role-Playing Game that lead to change

Role-playing Games have fundamental elements in their structure, regardless of the game system or setting, that are essential to the intervention. These elements enable the training of the social skills described previously, as outlined below (see **Table 3**):

**Table 3:** Elements of the Role-Playing Game that lead to change.

RPG Element	Driver of change
Character creation - stats, abilities, background	Recognition and debriefing of the player’s personality aspects projected in the creation of the character.
Fictional narrative with hooks and tasks	It keeps players motivated to pursue a goal and coordinate the group
Order of action - each player has to take actions in their own turn	It creates situation to train group coordination and civility
Collaborative game	As a collaborative intervention, it facilitates relationship-building and conflict management.
Unpredictability of Dice rolls	It promotes situations that help dealing with unexpected situations, frustrations and quick problem-solving ideas
Character progression - level/abilities progression	Conflict resolutions are rewarded with new abilities/powers → positive reinforcement
Narrative with emotional and bonding possibilities	It fosters the establishment of new connections and relationships, while also providing opportunities to cultivate empathy through various scenes.
Role Playing	Congruence, self-exploration, simulation, acting-out
Structured experience with rules and dynamics	Basis of acting-out, re-signification with a controlled environment.

##### Character creation (statistics, abilities, background)

Once the intervention setting/scenario is defined, participants are tasked with creating a character sheet detailing their abilities, skills, and statistics. This process is facilitated by *The Guide* and the *Meta Guide*, particularly concerning the system rules. However, this opportunity is also utilized to explore the participants’ personality traits as projected onto their characters and to promote insight during the debriefing session.

##### Fictional narrative with hooks and tasks

The RPG session revolves around a storyline that directs player interactions, offering prompts to encourage engagement, maintain participant motivation, and pursue a common goal. These hooks frequently tie into the backgrounds created by the participants, thereby enhancing the immersion necessary for the intervention’s success.

##### Order of action (each player has to take actions in their own turn)

In most RPGs, there arises a need to organize participants’ actions into turns, typically occurring during combat scenes but also proving useful in non-combat situations. This system prompts participants to consider their next actions and await their turn to announce them, while also requiring attentiveness to their colleagues’ turns. Consequently, this element fosters group coordination and civility.

##### Collaborative game

While competitive games offer opportunities for social skills training, RPGs are inherently collaborative, providing participants with the chance to cultivate relationships and practice conflict management skills.

##### Unpredictability of Dice Rolls

The randomness inherent in dice rolling can lead to significant successes or failures for characters in various tasks. While this mechanic adds an element of fun, it also prompts participants to adapt to unexpected outcomes and challenges. It encourages the group to think on their feet, fostering creativity and problem-solving skills.

##### Character progression - level/abilities progression

When certain tasks are completed or participants reach specific points in the narrative, their characters gain new abilities or enhance their skills. This progression, commonly referred to as “leveling up,” rewards successful conflict resolution and task completion by empowering characters to tackle more challenging obstacles. This mechanism serves as positive reinforcement for collaboration among participants in overcoming scenario challenges.

##### Narrative with emotional and bonding possibilities

RPGs serve as potent instruments for crafting immersive fictional scenarios wherein participants can forge emotional connections with non-player characters (NPCs) created by The Guide, as well as with themselves and fellow participants. These opportunities are vital components for fostering participants’ heightened awareness in recognizing others’ feelings and honing empathy skills.

##### Battle system

Sometimes players will encounter challenges that can trigger some kind of combat. The battles are organized in turns, where a player must plan and execute his limited actions and then wait for his teammates to proceed with the plan. Combat requires extensive teamwork, strategic thinking, and tactical maneuvers from players, all while maintaining constant communication. Additionally, it serves as a training ground for developing skills such as divided attention, inhibitory control, maintaining eye contact, flexibility, and patiently waiting for one’s turn.

##### Puzzles

Game features numerous puzzles organized by *the Guide* that require players to be highly organized, with extensive planning, logical reasoning (and deductive skills), cognitive flexibility, and teamwork to solve it.

##### Solving Problems with countless (and customizable) outcomes

Whenever players complete a main or secondary quest, the Guide determines the possible outcomes, which can vary from in-game rewards to direct impacts on the game’s environment/narrative. This provides an excellent opportunity for players to reflect on their decisions and observe their impact, allowing them to re-evaluate the group’s behavior, thoughts, and propose alternatives and new strategies.

#### Expected health outcomes

- Participants are able to discuss different treatment plans, listen to divergent opinions, express themselves clearly and work together towards a common objective to promote the best medical conduct;
- Participants will have better understanding of their own role in the team and be more comfortable to ask for help and share tasks;
- Professional and interpersonal relationships among staff members will enhance as they share meaningful experiences during the RPG sessions;
- Participants will adopt a more empathetic approach toward their colleagues and patients.;
- Enhanced well-being and mental health scores through the development of social skills.

These assessment tools can be quantitative, qualitative, or a combination of both (quali/quantitative). To ensure accurate data collection, assessments should be conducted at various points in time:

1) Pre-intervention: The questionnaires and scales must be administered before the intervention;
2) Mid-intervention: If the proposed intervention spans multiple RPG sessions, it is important to collect data between sessions;
3) Post-intervention: After the final debriefing session.

This information must be collected from all the individuals involved in the intervention: the participants (players), *The Guide* and the *Meta Guide*.

### Step 3. Program design

#### Intervention design

A critical skills TTRGP session follows a specific timeline, opening with behavioral assessment followed by 1 to 2 hours of gameplay sessions and 30 minutes to 1 hour of debriefing.

##### A) Session structure

###### Opening and rapport (15 minutes)

In the opening session the therapist will introduce the players to the system to be used in the game and its rules, participants will also be required to answer questionnaires to assess social skills and overall mental health. Limits to the narrative and personal preferences will also be discussed in this session in order to ensure a pleasurable experience and to identify themes that should not be discussed in the intervention for the whole group. In the following sessions a brief rapport will be conducted at the beginning of the activity to assess technical difficulties and rules misunderstandings.

###### Behavioral assessment and setting goals (15 minutes)

Following the initial rapport, the Guide and Meta Guide will ask each participant for a behavior presented in the last session, then discuss with the group if such behavior was adequate for the scene presented and what other course of action could have been used to achieve the same goal. Once all participants share a behavior/scene, the therapist then assesses which social skills based on the model proposed by Del Prette and Del Prette (39) should be improved in the following sessions.

###### Role-playing game intervention (1h – 1h30 hours)

The game is composed of social, combat and exploratory scenes that create an overall narrative. Social scenes are adapted according to the social skills defined as goals for the session. The narrative can be adapted to whatever setting/system the group is interested in. However, the core narrative will focus on the social skills that were deemed to be underperforming based on the questionnaires and interviews conducted in the opening session. The sessions can present three discussion triggering scenarios. The first one is when the group successfully applies and develops social skills and cooperative problem-solving strategies when dealing with narrative events. The second one is when the group uses the social skills, they already have to solve narrative conflicts, but also engages in aggressive and/or antisocial behaviors. And a third one is when the group relies only on aggressive and/or antisocial strategies for problem solving.

###### Debriefing (30 minutes to 1 hour)

Once the game session is over, the therapist will then select a scene and initiate a discussion, starting by the triggering scenario option chosen by the group and why said path was selected. Then alternative hook options will be presented to the group for debate, to assess if the chosen course of action was adequate or inadequate and how those other hooks could be achieved via narrative development.

###### Out of session insight

By the end of the session *The Guide* will remind the participants to answer in their homes a scene form, where they will present a specific scene from that day’s session that impacted them somehow and state the reasoning behind their actions as well as other courses of action that they believe could be explored. The scenes fed to this form will guide the rapport at the beginning of the next session.

##### B) Intervention mechanisms

###### The Guide and Meta-Guide

The intervention team will evaluate the needs of the group prior to the intervention, define the intervention program goals and structure the intervention. The primary narrator, named as *The Guide* will lead the storytelling and propose the situations for role playing, according to the programmatic goals. It is the primary narrator’s role to identify potential scenarios or adapt the session to focus on the specific goal/outcome of that training session. For instance, it is the Guide’s to build a narrative for a session planned to elicit social integration. If the session builds away from that goal, the primary narrator will lead the scene by inserting elements such as plot twists or other characters to interact.

The second narrator, (*Meta Guide*), will have multiple support roles to the implementation of the intervention. One role is to function similar to the concept of an auxiliary ego, as suggested by the literature on psychodrama. Introduced as a player in the game, the *Meta Guide* will be aware of the story narrative and the programmatic scene goals. Situated as players, the *Meta Guide* acts as a support to the primary narrator. In this role, they can emphasize aspects of the narrative to guide the scenes to the programmatic goals or function as a side counselor to support the debriefing or control emotional reactions to the story. The *Meta Guide* will also support the program development and its implementation.

###### Narrative

The narrative is the conceptual background that creates a context for the social, combat and exploration scenes without depending on a specific theme and or time age. Therefore, any narrative can be adapted to fit in the intervention model and any scene can be used as a proxy to stimulate a social skill with this method. Since the method is based on simulating real life scenarios, the narrative provides contextual details that help the participants to empathize and act from the character’s perspective.

###### Scenes build to make up the narrative

A role-playing game is based on a plethora of scenes, be them a social interaction between the participants and a non-playable character (NPC) or a combat encounter. This method creates a framework so that a scene can be adapted to reinforce a specific social skill (if that is the objective being worked upon). In order to categorize the assortment of scenes that are bound to happen during the intervention, a scene library will also be created to allow easy browsing and selection of scenes to be used in future applications (item d).

###### Game elements

*Non-playable characters (NPC’s):* Characters that populate the fictional world where the narrative takes place, they usually serve to further develop scenes in order to create triggering scenarios where a target skill can be developed.

*Narrative cues*: Scenes that lead to another scene or have a great impact in the world as a whole. They serve to bind the narrative together and allow for a steady pace of progression for the participants, making the experience more fluid and natural.

*Chance*: RPGs rely on a plethora of mechanics to ensure pseudo randomized outcomes. This means that while each character’s individual features have influence in whether they succeed or not in a specific task, there is always a random factor as well (represented by the dice roll). One such mechanic is the “*ability check*”, which is requested by *The Guide* in order to determine if a character can use one of its skills successfully.

*Systems*: TTRPG is a broad term used to define a genre of board/narrative games. Different types of RPGs exist in the market, aimed at a variety of audiences and with different themes (**Table 4**). This method is non-system dependent, meaning that most game system available in the market can be adapted to work as an intervention.

**Table 4:** Different TTRPGs systems.

System	Theme	Therapeutic Application Suggestion
Dungeons and Dragons/Pathfinder	Medieval fantasy	Group management skills and conflict management
Call of Cthulhu	Horror	Stress management and empathy
Kids on Bikes/Tales from the Loop	Realistic/suspense	Assertiveness and effective communication
Starfinder	Science fiction	Conflict management and group management

*Scenes library*: We are building a scenes library that will provide *The Guide* with the context, conflict, and elements necessary to have the participants training the required social skill (**Table 5**). These situations can be inserted in any setting, and they are not restricted to a certain system or scenario. Below we present two examples.

**Table 5:** Example of social skill and scene relation.

Social Skill	Scene	Example
Conflict Management	“Divergent Paths”	The participants are presented with two ominous paths to choose from. They must engage in discussion and collectively decide which path to follow, thereby accepting the consequences together.
Empathy	“Understanding the enemy’s motivation ”	The group finds out that the enemy is fighting to save his/her family. The participants engage in a debate regarding this newly discovered information.

##### C) Interventional strategies

There are several strategies that can be used as part of the intervention. These strategies are not mandatory, but they can provide interesting tools to help *The Guide* during the RPG session:

###### Fast-Forwards

Participants can foresee the consequences of their actions. *The Guide* describes them to the participants so they can have the knowledge of the future that their characters probably wouldn’t have at that moment.

###### Do-over

If an action or decision results in an unsatisfactory outcome, *The Guide* can allow the participants to turn back in time and do-over that particular situation. This is a useful tool if used in a correct context, but it could undermine the conflict of participants dealing with their choices that generate unsatisfactory outcome.

###### X-Card

Even if the participants and *The Guide* decide together which situations are a trigger for psychosocial stress and should be left out of the gaming session beforehand, there is always the chance that some other issue might come up during the game. It’s important that participants have the means to tell the group and *The Guide* that they are uncomfortable and would rather skip that scene without having to interrupt the narrative abruptly. The “*X-Card*” (a virtual or physical sign that can be shown to the group at any moment) allows *The Guide* to quickly jump a scene that can be potentially triggering, and the triggering scene will then be discussed in the debriefing.

###### In-game debrief

Some conflicts may arise during the session that cannot be solved in-character. Some emotional situations may have a participant in need of a quick break. These situations may require *The Guide* to do an in-game debrief and use this opportunity to enhance the possibilities of the intervention.

###### Collective problem-solving

Depending on the situation, one or more player characters might struggle to solve a problem. This strategy allows the group of participants (not the characters) to collectively discuss the problem and solve it as a group.

###### Pause

Sometimes the in-game time flow feels too fast for the participants to organize their actions or to make a decision. When the participants feel overwhelmed by the game events it might be useful to be able to “pause” the narrative and allow the group to gather their thoughts and organize them.

###### Character switching

During the activity, participants will have to change characters amongst themselves and/or to control an NPC a few times, making them see situations in different perspectives and understand the competences and responsibilities of different roles. Character switching will Possibly this activity evokes aspects of social skills, such as perspective taking and empathy in general (44).

###### Narrative negative and positive outcomes

As a real-life simulation, the game will promote the presentation of positive and negative consequences, with the participant having to deal with them in order to be able to keep playing and to understand what is happening. It is up to *The Guide* to ensure that the session will have a range of outcomes to character actions based on the story, environment, game rules, and elements of chance. The guide can support players in engaging with these outcomes and managing their personal and group responses to them in a functional way, as well as conducting the game in the best way to deal with the frustrations of the participants in face of negative consequences, as well as with the exacerbation of excitement of the participants in face of positive consequences, which can be negative for the rest of the group.

### Step 4. Protocols, materials and tools

#### Intervention Start

The initial session will serve as presentation of the intervention model as well all the components in which the game will rely on. The rule of the chosen game will be introduced, alongside a character creation tutorial, participants will also partake in a quick guide to using physical or digital dice and how to navigate the online website in cases where the sessions are online. Once all players are introduced to the rules and instruments, a role-playing session will take place where characters can introduce themselves and present others with each character’s backstory and motivations. The guide will also introduce the basic storyline for the narrative and inquire the player in regard to any discomfort with the proposed narrative, taking notes if needed to alter the narrative components in case any players present a discomfort. At the end of this initial briefing, players will be required to answer the mental health and social skills protocols described in **Table 6**. And will also receive the player´s journal for the following sessions.

**Table 6:** Protocol of the intervention.

Session Element	Time	Goals	Supporting Document
1 Introduction to the adventure and bonding	1 to 2 sessions	Creating a bond between participants and introducing the campaign setting, alongside with rules and the scope of the narrative.	Mental health screening scales (GAD-7, PHq-9, AUDIT, IHS-2 and ASRS-18), introduction to the players journal and first assignments.
2 Introduction to the first therapeutic narrative objective and conflict building	4 to 5 sessions	Based on results from the mental health screening scales, a narrative will be selected to match the therapeutic needs of the group. In these sessions the group will be presented with said conflict and will have to work as a group towards a resolution.	The player’s journal will be utilized to record and review the player’s perceptions and inputs regarding the proposed challenges. Additionally, the journal will serve as an emotional log, allowing players to document how various situations made them feel during and after the session.
3 Feedback collection and role-play intermission	1 to 2 sessions	These sessions are designed to encourage players to reflect on the challenges they have just completed, reviewing their choices and behaviors. *The Guide* should introduce a character to facilitate role-play discussions, extracting insights into each player character’s thought process and understanding which information each one deemed most important to achieve the proposed objective.	The players’ journals will contain a feedback sheet where players can highlight scenes or conflicts that affected them beyond the session setting. They can also indicate if they regret any course of action and suggest how that action could be undone or if there were any alternatives. This feedback sheet will also enable players to speculate on the consequences of their actions in the narrative setting.
4 Closing remarks and Follow-up	1 to 2 sessions	These sessions will conclude the narrative path chosen by *The Guide* at the beginning of the intervention, providing closure to the individual stories of the characters as well as an ending to the overall narrative. Players will be asked to provide background for their characters’ ending, expressing their goals and wishes and explaining why they chose them for the character.	The players will then complete the mental health screening scales again for post-intervention data collection and submit their player’s journals.

#### Narrative proposal and conflict presentation

Once all players are introduced to the gaming system, alongside the player´s journal and finished training with dice and character sheets the narrative can then start. In this step, the *guide* must customize the narrative to fit the group profile measured in the previous step through scales and questionnaires, a *Guide* can create an engaging scenario of its own volition if he/she has enough experience, however for new *Guides* a scene library has been created, containing a multitude of scenes and which skills/needs are addressed in each one of then, alongside with its outcomes, and we advise its usage until the *Guide* can create custom scenarios. During these sessions, players will be required to take notes on the narrative events that they believe would be important for their characters and if said event caused an emotional response in themselves. At the session’s end, all players will be required to report an event, stating the outcome lived in the narrative, what was the expected outcome, why the expected outcome was not achieved and what could’ve been done differently.

#### Feedback and Intermission

Once the intervention narrative is concluded, the *Guide* will promote an intermission session, where the consequences of the players choices will be presented out of character, the intermission will also be used to discuss alternate courses of action the party could have taken and its consequences. All players will be requested to explain their characters motivation and overall mental state when making choices during the narrative and explain why they perceived the scenario in said way. Players will also be encouraged to provide feedback and suggestions regarding narrative plots and possible conflicts to arrive in following sessions. At the end of this session, players will be asked to answer the feedback form present in the player´s journal.

#### Closing remarks and follow-up

The *Guide* will proceed to end the narrative storyline; each player will be required to provide a foreground to how they see their characters journey ending and why such path would be important for the character. Players will also be asked to report on the experience of playing said character and if emotional or behavioral conflict during gameplay. Finally, players will be required to answer a follow-up questionnaire to the mental health screening they answered at the start of the intervention. Then the player´s journals will be collected for content analysis and the intervention ends.

#### Products

The intervention proposal serves as a framework for applying CBT therapeutic guidelines in a gamified setting, to provide further scientific evidence and support to those attempting to incorporate this method in their practice a streaming service alongside with two podcasts will be made available.

#### Streaming

The streaming service will be provided via twitch, at https://www.twitch.tv/iniciativacriticalskills, where weekly sessions will be streamed alongside the discussion sessions for each episode. Older episodes and discussions can be found at https://www.youtube.com/@CriticalSkills. Streaming episodes contain only mock sessions where the researchers demonstrate the methods.

#### Podcasts

Two podcasts were created to provide scientific background and feedback to incoming practitioners, both are available in multiple podcast streaming services, but mainly on Spotify. One is called *Ability Check* and presents a scientific focused review of techniques and studies that used serious games in their methods, silverling potential pitfalls and highlighting promising studies for replication and further development.

### Step 5. Example of program implementation

In order to make the application of this program tangible, below we exemplify the implementation of the program carried out by our group. The work presenting the experiment and the result in detail is in writing.

#### An example of using the Critical Skill model with psychiatry residents

This program was carried out in a collaboration with the Department of Mental Health - Santa Casa de São Paulo Medical School, Brazil. Thirteen psychiatrist residents from the first to third years of residence participated in this program. The process of developing this intervention and its structure according to the Critical Skills methodology will be described below. To design this intervention and its implementation we followed the Intervention Mapping (33) and the Consolidated Framework for Implementation Research (32) frameworks.

#### Defining the problem: mental health and resilience training during COVID-19 pandemic

Medical residency is recognized as a highly stressful period, with a higher prevalence of depression (3 to 16% higher than the general population) (45). Factors contributing to this stress include intense emotional demands, such as sleep deprivation, constant exposure to suffering patients, lack of autonomy, and a fast-paced work environment. Additionally, issues such as burnout and alcohol abuse have been reported during medical training (46–55). These challenges were further exacerbated by the social isolation measures and lifestyle adjustments necessitated by the COVID-19 pandemic (56). In the particular case of the psychiatry residency program at the Santa Casa de São Paulo General Hospital, mental health concerns among residents were of particular concern to medical directors and preceptors. With new cohorts of residents arriving during the pandemic, challenges such as the need to treat COVID-19 patients, limited physical interaction with peers and mentors, and difficulties in adapting to a new city without proper onboarding experiences became apparent. These issues, identified during the needs assessment phase through interviews with residents, medical directors, and preceptors, led us to explore the use of virtual tabletop RPG experiential learning training as a potential solution. At the time, the research team comprised a psychiatry preceptor with expertise in resident training, RPG, and group therapy. Together, we collaborated with medical directors and residents to establish an intervention program later named the “Critical Skills Initiative,” an RPG-based experiential learning program aimed at developing social skills and promoting mental health reintegration. The theoretical framework of social skills training was chosen due to its association with positive mental health outcomes, while the use of serious games, targeting outcomes beyond mere gameplay, was also linked to mental health benefits. By engaging with key stakeholders in the psychiatry residency program, we identified the need for an intervention focusing on social integration and mental health resilience through the enhancement of social skills.

#### Setting

Santa Casa de São Paulo is a tertiary care, academic healthcare system located in São Paulo, Brazil. Serving as a referral center for the entire metropolitan area of São Paulo, which encompasses over 20 million people, it also provides healthcare services to patients from across the entire state and country. Santa Casa hosts numerous Medical Residency Programs, boasting a cohort of over 700 medical residents and 300 post-graduate students. Additionally, it serves as an internship site for more than 1,000 healthcare undergraduate students from Santa Casa de São Paulo Medical School.

#### Program outcomes, objectives and logic model of change.

The Critical Skills program was implemented for medical residents with the aim of enhancing their social cognition repertoire, promoting social integration during periods of isolation, and bolstering mental health resilience. **Table 7** illustrates the logic model outlining how our intervention was structured based on the identified social skills needs, along with the targeted outcomes.

**Table 7:** Intervention planning logic model applied to the Critical Skills program.

Social Skills	Daily Determinants	In-game Dynamics	Intervention Outcomes
**Conflict management and resolution**	Discuss different treatments for a patient	Discuss various in-game plans to solve a problem.	Agree to adhere to the plan selected by the group through voting.
**Effective communication**	Having ideas and thoughts that are not clearly understood by the staff, which leaves room for misinterpretation.	Player characters must effectively communicate their ideas to both their colleagues and non-player characters (NPCs).	Ideas and thoughts are communicated in a manner that ensures full comprehension by all staff members.
**Civility**	Conversations, particularly in video conferences, often lack efficiency as it is challenging to discern the appropriate timing for speaking and allowing others to speak without interruption.	The group of player characters utilizes their initiative rolls and other cues provided by *The Guide* to ensure that everyone has the opportunity to take their actions and express themselves.	Subjects demonstrate increased awareness of when to initiate communication and respond to it. They exhibit greater respect and ensure that everyone is listened to attentively.
**Relationship management**	Medical residents frequently encounter challenges in establishing new relationships within a different environment. This difficulty can contribute to an uptick in mental health issues, including depression, anxiety, and substance abuse.	The RPG intervention facilitates the creation of scenarios where players can forge strong bonds and nurture healthy relationships. The playful aspect of this intervention is pivotal in fostering such connections.	The relationships built in-game foster the development of healthy professional connections
**Assertiveness**	Feeling unable to question, express disagreement, communicate with figures of authority, and/or handle criticism can result in significant ideas not being shared and discussed.	Situations in the RPG scenario necessitate that player characters demonstrate assertiveness to effectively solve problems.	Subjects feel more empowered to share their ideas and opinions
**Empathy**	Failure to understand and empathize with the feelings of others can adversely affect relationships between colleagues, medical staff, and patients.	The feelings of both the players and their player characters, as well as the NPCs created by *The Guide*, are deliberated upon during the debriefing.	Subjects make a more conscious effort to assess the feelings of others
**Group management**	Tasks are not properly completed due to the staff’s inability to effectively coordinate the group and delegate tasks	Each character class serves a distinct function, and it is essential for the group to understand each one’s role in order to achieve a positive outcome.	The group acknowledges their strengths and weaknesses, delegates tasks, and manages goals effectively.

With the intervention goals defined, we also discussed what would be measured and how to monitor the progress and the impact of the intervention. Before the start of the program, we planned to conduct a focus group to evaluate the residents’ social skills and current status of social integration. During this session, we also assessed a set of core mental health metrics, listed in **Table 8** below. The results from the focus groups and questionnaire assessments served as guides to plan the TTRPG campaign. We also intended to collect the same metrics during and after the intervention sessions.

**Table 8:** Outcome measures and assessment methodology.

Outcome	Assessment Methodology	Timeline
Social Skills	Social Skills Inventory (41)	Before and after the intervention
Personality	Big Five Reduced Markers (57)	Before the intervention
Mental health	PHQ-9 (58)	Before, during and after the intervention
Alcohol use	AUDIT (59)	Before the intervention
Psychological well-being	Psychological well-being scale (60)	Before and after the intervention
Needs assessment	Focus group	Before, during and after the intervention

#### Program design and production

The game used in the intervention was Dungeons and Dragons (D&D), an RPG created in 1974 by Gygax and Arneson (61). D&D is highly adaptable and can be customized for various purposes, requiring only dice and character sheets to play (26). Residents participated in weekly 3-hour sessions for 4 months. All sessions were structured according to 5 fundamental steps:

##### Rapport (10 minutes)

Before starting the sessions, participants gathered to discuss the previous session, making suggestions about the adventure, system, gameplay, and other technical aspects or group dynamics.

##### Task Discussions (20 minutes)

Every week, participants filled out forms to document the game situations they considered significant, along with the thoughts and behaviors that influenced their decision-making. Subsequently, they were asked to suggest alternatives and reflect on the consequences of their actions, as well as the quality of the outcomes.

##### Goal Setting (10 minutes)

Participants and the GM would set goals for the sessions, proposing alternative behaviors and new goals to be achieved within the game. From the discussion of the task, the GM developed an individual plan for the players according to the desired target abilities.

##### The game (90 minutes)

The system used was Dungeons and Dragons. The adventures comprised social situations designed to demand social skills that needed training.

##### Feedback (60 minutes)

After the adventure ended, the GM provided feedback to the players, highlighting ideas, behaviors, decision making and consequences, reminding them to fill out the forms for the next session.

#### Program implementation

We divided 13 medical residents from the Psychiatric Residency Program in our institution into 3 different groups based on their year in the program. Group A consisted of 3 males and 3 females with a mean age of 27 years; Group B had 2 males and 2 females with a mean age of 26 years, and Group C included 3 participants, 2 males, and 1 female, with a mean age of 24 years. All participants underwent interviews and clinical assessments for psychiatric disorders, primarily major depressive disorder and generalized anxiety disorder, with no clinically relevant symptoms detected. For this intervention, we selected the 5th edition of Dungeons & Dragons™ as the system, and our team created the scenario, also known as a “homebrew setting.” The narrative plot placed the participants’ characters in a small village on a quest to locate magical artifacts related to the legend of an evil necromancer. Additionally, the Meta Guide provided support in-game for the participants, although their role did not involve active problem-solving.

After the first session and by the end of all the subsequent ones, the participants were required to answer a questionnaire that made them reflect on the situations that they found challenging, interesting or somehow important. At the beginning of the next session *The Guide* would conduct a debriefing meeting for 30-60 minutes and address the situations listed by the participants focusing on the social skills training.

The narrative arc encompassed 08 episodes that were conducted in 12 to 16 sessions. During this period, the participants were expected to face many challenges as they unraveled the mystery behind the magical artifacts and the characters involved in the plot. The participants’ characters progressed from level 1 to level 6-8 (depending on the group) and were placed in scenes designed to allow them to practice their social skills. Examples of these scenes are listed below in **Table 9**:

**Table 9:** Example of scenes, descriptions and social skills targeted.

Scene	Description	Social Skill
The cursed staff	The participants retrieved a cursed staff, but it proved to be a wrong decision as the evil necromancer was now able to return to this world. They were accused of being his allies and had to defend themselves.	Effective communication
The choice between two leaders	The participants faced the daunting task of choosing a side in the impending battle, each option presenting its own set of advantages and disadvantages.	Conflict management and resolution
Character’s death	One of the participant’s characters died in combat. The group had to deal with the loss of a beloved one and rethink their battle strategy.	Relationship management and group management

#### Session example

##### Opening and rapport

The participants share some rules questions which are promptly answered by The Guide and the Meta Guide. All the participants agree that, at this point in the intervention (halfway through the narrative), there are neither technical difficulties nor significant misunderstandings of the rules to address. They do, however, discuss some personal difficulties to be more assertive in certain situations.

##### Behavioral assessment and setting goals

The participants agree that the last session’s major event was their colleague’s character death in a combat. They share their feelings of impotence and frustration for not being able to help him. *The Guide* asks them what actions they would have changed given the opportunity. The following topics are cited: a) be more prepared (provisions, weapons, etc.) for the battle; b) used different abilities and skills (“I could have casted a different spell in my turn”); c) planned a different strategy (“my character could have shielded him if I was closer”). After this assessment, *The Guide* asks the participant whose character died how he was feeling. He shares his feeling and states that he is glad about the way his character died being a hero and protecting his friends. No other situation was brought in this assessment.

The participants agree that their characters must go on and must reach the bottom of the narrative mystery. They decide to talk more clearly about their intentions in combat scenes to be more effective. It is important to note that a character death is an important event during a TTRPG session and could be interpreted as a trigger for negative emotions. In our cause, we had previously discussed with the group about character death as a game mechanism, and it was not deemed a concern. We suggest that Guides discuss this topic at the Opening and Rapport moments before bringing this to the narrative. Similarly, the debriefing session need to allow for the character to process the event. Lastly, as part of the goal setting, the player was able to choose a new character to continue the sessions.

##### Role-playing game intervention

Following the event of the character death, the Guide prepares a session that allows the players to enact their experiences with the character’s death, processing the event as characters as well as players.

This session began with the debriefing. First, we asked them to describe the scene from memory. We asked them how they felt, and they talked about frustration and guilt for not knowing how to make “correct” decisions. Then, the player whose character died said he was more impacted than he thought he would be by the death of a fictional character, but that he was feeling calm afterwards. The other players realized that they had also been sad, but that they were more concerned about the player who lost the character than he was himself We brought up this feeling of guilt and if they thought they should have acted differently. They talked about action economy, optimizing rounds, etc … Then we pulled out definitions of teamwork, communication.

Lastly, the players talked about dealing with frustrations. One player was frustrated because they had joined the group late, and shortly afterwards, their character died. They meant it as a “joke,” but it was also brought up for discussion. Then the players talked about how this event could either make players want to quit or increase their motivation. The players expressed a sense of obligation to see the journey through to the end in honor of the fallen character.

##### Debriefing

The participants joyfully protest against being left in a cliffhanger. *The Guide* presents some of the choices they made and the dilemmas they faced. They ask about the consequences of their choices, but *The Guide* opts to share only the information that will not impact the future sessions.

##### Out of session insight

By the end of the session, *The Guide* reminds the participants to answer the questionnaire during the week before the next session. The participants have to think about this session’s scenes and their actions and choices.

### Step 6. Program evaluation

Our intervention evaluation was designed to employ qualitative content analysis of medical residents undergoing social skills training with RPG, utilizing CFIR metrics as a framework. Upon completion of the intervention program, we reassessed the residents using the metrics outlined in **Table 9** and conducted focus groups for process evaluations. We also conducted ongoing evaluations, gathering written feedback from participants on a weekly basis through recall exercises, and documented intervention observations notes from *The Guide* and *The Meta-Guide*. The implementation research team would convene weekly meetings with *The Guide* and *The Meta-Guide* to review the feedback and intervention notes and discuss the implementation process. However, as the primary focus of the present work is to introduce the method rather than delve into the results obtained from its implementation in the described population, a separate article detailing the experiment and its results is currently being written.

## Discussion

The objective of this study was to devise a method/protocol utilizing Table-Top Role-Playing Games (TTRPGs) as an intervention tool to tackle mental health challenges, particularly focusing on social skills training. We employed the intervention mapping framework, amalgamating TTRPG and Cognitive Behavioral Therapy (CBT) principles. Additionally, we offer an illustrative application of the method, conducted concurrently by our team, to demonstrate its practicality and feasibility. Subsequently, we delve into a discussion concerning the rationale, viability, and clinical implications of the method, along with potential limitations.

### Justification of the method

As outlined in the introduction, gamified interventions have emerged as a burgeoning approach in mental health prevention. Their positive impacts on managing various clinical conditions stem from their ability to promote social skills (18–21). However, cost-effective alternatives like Table-Top Role-Playing Games (TTRPGs), which offer similar benefits to other game-based interventions, lack standardized methods, hindering replicability. Hence, TTRPGs hold promise as a playful alternative, incorporating gamified elements into the well-established Role-Playing technique to serve as an experiential method. With a structured adventure, it encourages experiential learning and facilitates the training of social skills. In this context, the method presented in this paper aims to create a protocol in six steps for developing an intervention via TTRPGs, guided by the Consolidated Framework for Implementation Research (CFIR).

Throughout these steps, we draw upon robust literature on social skills training (SST), cognitive behavioral therapy (CBT), and gamification. Thus, our method encompasses the objectives of SST, the strategies of CBT, and the dynamics of gamification through TTRPGs.

### Feasibility of the method

One crucial aspect that received considerable attention during the development and drafting of this method pertains to the integration of evidence-based effectiveness with the method’s practical applicability. Within the context of Social Skills Training (SST), certain digital techniques and approaches have demonstrated notable effectiveness, albeit often accompanied by substantial costs, whether financial or in terms of required infrastructure (22). This renders widespread implementation in society unfeasible. Conversely, numerous playful activities commonly employed in daily social, familial, and educational settings, while boasting low implementation costs, often lack empirical evidence regarding their efficacy as social skills intervention tools. Consequently, the creation of a structured instrument capable of amalgamating cost-effectiveness and evidence-based practice assumes paramount importance for clinical, educational, and public policy applications.

During its development, this method drew upon literature on Social Skills Training (SST), Cognitive Behavioral Therapy (CBT), and gamification, demonstrating ease of elaboration, implementation, and comprehension among participants. As outlined in step 5, the application of our method with psychiatry residents serves as a concurrent endeavor undertaken by our team to validate both the intervention’s impact on social skills and its feasibility. In a concurrent publication, currently in progress, we present qualitative findings derived from participant interviews and testimonials. In summary, our findings suggest that the method effectively enhances social skills, is readily implemented by *The Guide*, comprehensible to participants, and entails minimal implementation costs. Consequently, we assert that the method is indeed feasible for integration into clinical, educational, and public policy settings.

### Limitations

As main limitations of the method, as shown in **Table 1**, each element of TTRPGs has potential pitfalls, which need to be taken into account when designing clinical protocols and intervention strategies in future studies. Nonetheless, we understand that all these limitations can be mapped, studied, controlled, and managed in the clinical and/or experimental scenario.

In our pilots, one important variable identified when initiating the intervention strategy was the players’ prior familiarity with the 5th Edition Dungeons & Dragons TTRPG. Character creation with free-form options can be overwhelming for new and unfamiliar players. In later iterations of the intervention model, we introduced limited and pre-generated characters with options like spells and weapons already selected to facilitate newcomers’ understanding. Player attendance can also pose a challenge to the pacing of the narrative and the outcome of the intervention. Players with low attendance may miss relevant scenes in the narrative and become emotionally detached from other characters and players. Finally, we also perceived what is commonly known as “main character syndrome” (MCS) to be a detrimental factor when using TTRPG’s as an intervention model. MCS occurs when one player in the group begins to dominate the narrative to the extent of acting against other players’ consent. A player exhibiting MCS can be viewed as both a problem and an opportunity for the Guide, as this behavior can illustrate a lack of social skills, which the intervention aims to address. However, both *Guides* and *Meta Guides* must be aware of this pattern and intervene during sessions both in role-play and in the feedback sessions, to prevent it from occurring and to help the player self-regulate their behaviors to maintain an engaging experience for the other participants. Otherwise, engagement by other players may decrease, potentially leading to dropout.

## Conclusion and future directions

The present work aimed to develop a method/protocol to use TTRPG as an interventional tool to address the mental health burden, particularly focusing on social skills training, through the integration of TTRPG and CBT within the intervention mapping framework. The resultant method is presented as structured, evidence-based, easy to implement, cost-effective, and thus feasible. Future research endeavors and mental health professionals seeking a structured and instructional instrument for protocol development will find support in the method proposed herein.

## Data availability statement

The original contributions presented in the study are included in the article/supplementary material. Further inquiries can be directed to the corresponding author.

## Ethics statement

This study has been reviewed and approved by the ethics committee from the Santa Casa de Misericórdia de São Paulo (CAAE: 105 40539920.0.0000.5479). All participants provided their written consent prior to entering the program.

## Author contributions

VO: Writing – original draft, Project administration, Data curation, Conceptualization. RN: Writing – review & editing, Methodology, Formal Analysis, Conceptualization. JP: Writing – review & editing. PN: Writing – review & editing. TR: Writing – review & editing. RCh: Writing – review & editing. JG: Writing – review & editing. LM: Writing – review & editing. JN: Writing – review & editing, Writing – original draft.

## References

[1] Rehm J Shield KD . Global burden of disease and the impact of mental and addictive disorders. Curr Psychiatry Rep. (2019) 21:10. doi: 10.1007/s11920-019-0997-0 30729322

[2] Center for Behavioral Health Statistics and Quality . Behavioral health trends in the United States: Results from the 2014 National Survey on Drug Use and Health. (2015). Available at: https://www.samhsa.gov/data/sites/default/files/NSDUH-FRR1-2014/NSDUH-FRR1-2014.pdf.

[3] World Health Organization . Prevention of mental disorders: effective interventions and policy options summary report. Geneva: World Health Organization. (2004).

[4] Fusar-Poli P Salazar de Pablo G De Micheli A Nieman DH Correll CU Kessing LV . What is good mental health? A scoping review. Eur Neuropsychopharmacology: J Eur Coll Neuropsychopharmacol. (2020) 31:33–46. doi: 10.1016/j.euroneuro.2019.12.105 31901337

[5] Ezawa ID Hollon SD Robinson N . Examining predictors of depression and anxiety symptom change in cognitive behavioral immersion: observational study. JMIR Ment Health. (2023) 10:e42377. doi: 10.2196/42377 37450322 PMC10382949

[6] Kasari C Shire S Shih W Almirall D . Getting SMART about social skills interventions for students with ASD in inclusive classrooms. Exceptional Children. (2021) 88:26–44. doi: 10.1177/00144029211007148

[7] Płatos M Wojaczek K Laugeson EA . Effects of social skills training for adolescents on the autism spectrum: a randomized controlled trial of the polish adaptation of the PEERS® Intervention via hybrid and in-person delivery. J Autism Dev Disord. (2023) 53:4132–46. doi: 10.1007/s10803-022-05714-9 PMC9399988 36001196

[8] Muroi F Tao X Han T . A Study on the Effect of Gamification on Alleviation Anxiety Levels of the Elderly in China. Cham/Switzerland: Springer Nature. (2020) pp. 329–42. doi: 10.1007/978-3-030-50249-2_24.

[9] Lukas CA Eskofier B Berking M . A gamified smartphone-based intervention for depression: randomized controlled pilot trial. JMIR Ment Health. (2021) 8:e16643. doi: 10.2196/16643 34283037 PMC8335612

[10] George D Jameson JP Michael K Yarbrough J George D . Assessing the efficacy of a self-administered treatment for social anxiety in the form of a gamified mobile application: A pilot study. J Technol Behav Sci. (2021) 6:124–36. doi: 10.1007/s41347-020-00158-3

[11] Rasouli S Gupta G Nilsen E Dautenhahn K . Potential applications of social robots in robot-assisted interventions for social anxiety. Int J Soc Robotics. (2022) 14:1–32. doi: 10.1007/s12369-021-00851-0 PMC8787185 35096198

[12] Baranowski T Buday R Thompson DI Baranowski J . Playing for real. Am J Prev Med. (2008) 34:74–82.e10. doi: 10.1016/j.amepre.2007.09.027 18083454 PMC2189579

[13] DeSmet A Van Cleemput K Bastiaensens S Poels K Vandebosch H Malliet S . Bridging behavior science and gaming theory: Using the Intervention Mapping Protocol to design a serious game against cyberbullying. Comput Hum Behav. (2016) 56:337–51. doi: 10.1016/j.chb.2015.11.039

[14] Pinón-Blanco A Vergara-Moragues E Fernández-Martínez R Gutiérrez-Martínez O Alvarez-Counago M Martínez-Reglero C . Effectiveness of the “Trisquel” board game intervention program for patients with schizophrenia spectrum disorders. Actas Espanolas Psiquiatria. (2020) 48:209–19. 33210279

[15] Estrada-Plana V Esquerda M Mangues R March-Llanes J Moya-Higueras J . A pilot study of the efficacy of a cognitive training based on board games in children with attention-deficit/hyperactivity disorder: A randomized controlled trial. Games Health J. (2019) 8:265–74. doi: 10.1089/g4h.2018.0051 30653355

[16] Kim B Lee D Min A Paik S Frey G Bellini S . PuzzleWalk: A theory-driven iterative design inquiry of a mobile game for promoting physical activity in adults with autism spectrum disorder. PloS One. (2020) 15:e0237966. doi: 10.1371/journal.pone.0237966 32911501 PMC7482920

[17] Litvin S Saunders R Maier MA Lüttke S . Gamification as an approach to improve resilience and reduce attrition in mobile mental health interventions: A randomized controlled trial. PloS One. (2020) 15:e0237220. doi: 10.1371/journal.pone.0237220 32877425 PMC7467300

[18] Bochennek K Wittekindt B Zimmermann S-Y Klingebiel T . More than mere games: a review of card and board games for medical education. Med Teacher. (2007) 29:941–8. doi: 10.1080/01421590701749813 18158669

[19] Hamari J Koivisto J Sarsa H . (2014). Does gamification work? – A literature review of empirical studies on gamification, in: 2014 47th Hawaii International Conference on System Sciences, . pp. 3025–34. doi: 10.1109/HICSS.2014.377

[20] Sardone NB Devlin-Scherer R . Let the (Board) games begin: creative ways to enhance teaching and learning. Clearing House: A J Educ Strategies Issues Ideas. (2016) 89:215–22. doi: 10.1080/00098655.2016.1214473

[21] Koivisto J Hamari J . The rise of motivational information systems: A review of gamification research. Int J Inf Manage. (2019) 45:191–210. doi: 10.1016/j.ijinfomgt.2018.10.013

[22] Gentry SV Gauthier A L’Estrade Ehrstrom B Wortley D Lilienthal A Tudor Car L . Serious gaming and gamification education in health professions: systematic review. J Med Internet Res. (2019) 21:e12994. doi: 10.2196/12994 30920375 PMC6458534

[23] Peracchia S Curcio G . Exposure to video games: effects on sleep and on post-sleep cognitive abilities. A sistematic review of experimental evidences. Sleep Sci (Sao Paulo Brazil). (2018) 11:302–14. doi: 10.5935/1984-0063.20180046 PMC6361300 30746049

[24] Fortes LS Lima-Júnior Dd Gantois P Nasicmento-Júnior JRA Fonseca FS . Smartphone use among high level swimmers is associated with mental fatigue and slower 100- and 200- but not 50-meter freestyle racing. Perceptual Motor Skills. (2021) 128:390–408. doi: 10.1177/0031512520952915 32867593

[25] Rivers A Wickramasekera IE Pekala RJ Rivers JA . Empathic features and absorption in fantasy role-playing. Am J Clin Hypnosis. (2016) 58:286–94. doi: 10.1080/00029157.2015.1103696 26675155

[26] Clarke S Arnab S Morini L Heywood L . Dungeons and dragons as a tool for developing student self-reflection skills. In: Games and Learning Alliance. Springer Cham (2019). p. 101–9.

[27] Blackmon WD . Dungeons and dragons: the use of a fantasy game in the psychotherapeutic treatment of a young adult. Am J Psychother. (1994) 48:624–32. doi: 10.1176/appi.psychotherapy.1994.48.4.624 7872422

[28] Daniau S . The transformative potential of role-playing games—: from play skills to human skills. Simulation Gaming. (2016) 47:423–44. doi: 10.1177/1046878116650765

[29] Wright JC Weissglass DE Casey V . Imaginative role-playing as a medium for moral development: dungeons & Dragons provides moral training. J Humanistic Psychol. (2020) 60:99–129. doi: 10.1177/0022167816686263

[30] Varrette M Berkenstock J Greenwood-Ericksen A Ortega A Michaels F Pietrobon V . Exploring the efficacy of cognitive behavioral therapy and role-playing games as an intervention for adults with social anxiety. Soc Work Groups. (2023) 46:140–56. doi: 10.1080/01609513.2022.2146029

[31] Abbott MS Stauss KA Burnett AF . Table-top role-playing games as a therapeutic intervention with adults to increase social connectedness. Soc Work Groups. (2022) 45:16–31. doi: 10.1080/01609513.2021.1932014

[32] Damschroder LJ Aron DC Keith RE Kirsh SR Alexander JA Lowery JC . Fostering implementation of health services research findings into practice: a consolidated framework for advancing implementation science. Implementation Sci. (2009) 4:50. doi: 10.1186/1748-5908-4-50 PMC2736161 19664226

[33] Bartholomew Eldrigde LK Markham CM Ruiter RAC Fernàndez ME Kok G Parcel GS . Planning health promotion programs: An Intervention Mapping approach. 4th ed. San Francisco: Wiley (2016).

[34] Bartholomew LK Parcel GS Kok G . Intervention mapping: A process for developing theory and evidence-based health education programs. Health Educ Behav. (1998) 25:545–63. doi: 10.1177/109019819802500502 9768376

[35] Kirk MA Kelley C Yankey N Birken SA Abadie B Damschroder L . A systematic review of the use of the Consolidated Framework for Implementation Research. Implementation Sci. (2015) 11:72. doi: 10.1186/s13012-016-0437-z PMC4869309 27189233

[36] Othlinghaus-Wulhorst J Hoppe HU . A technical and conceptual framework for serious role-playing games in the area of social skill training. Front Comput Sci. (2020) 2:28. doi: 10.3389/fcomp.2020.00028

[37] Alsawaier RS . The effect of gamification on motivation and engagement. Int J Inf Learn Technol. (2018) 35:56–79. doi: 10.1108/IJILT-02-2017-0009

[38] Bowman SL . Immersion and shared imagination in role-playing games. In: Role-Playing Game Studies. London: Routledge (2018). p. 379–94.

[39] Del Prette A Del Prette Z . Competência Social e Habilidades Sociais: Manual teórico-prático. Petropolis: Vozes (2019).

[40] Del Prette A Del Prette Z . Psicologia das Habilidades Sociais: terapia, educação e trabalho. Petropolis: Vozes (1999).

[41] Del Prette A Del Prette Z . Psicologia das relações interpessoais e Habilidades Sociais: vivências para o trabalho em grupo. Petropolis: Vozes (2001).

[42] Del Prette A Del Prette Z . A importância das tarefas de casa como procedimento para a generalização e validação do treinamento de Habilidas Sociais. In: Primeiros passos em análise do Comportamneto e Cognição. Santo André: Esetec (2005). p. 67–74.

[43] Leahy R . Cognitive therapy techniques: a practitioner’s guide. New York: The Guilford Press (2017).

[44] Iannotti RJ . Effect of role-taking experiences on role taking, empathy, altruism, and aggression. Dev Psychol. (1978) 14:119–24. doi: 10.1037/0012-1649.14.2.119

[45] Levy AB Nahhas RW Sampang S Jacobs K Weston C Cerny-Suelzer C . Characteristics associated with depression and suicidal thoughts among medical residents: results from the DEPRESS-ohio study. Acad Psychiatry: J Am Assoc Directors Psychiatr Residency Training Assoc Acad Psychiatry. (2019) 43:480–7. doi: 10.1007/s40596-019-01089-9 31290011

[46] Fahrenkopf AM Sectish TC Barger LK Sharek PJ Lewin D Chiang VW . Rates of medication errors among depressed and burnt out residents: prospective cohort study. BMJ. (2008) 336:488–91. doi: 10.1136/bmj.39469.763218.BE PMC2258399 18258931

[47] Oreskovich MR . Prevalence of alcohol use disorders among American surgeons. Arch Surg. (2012) 147:168. doi: 10.1001/archsurg.2011.1481 22351913

[48] de Oliveira GS Chang R Fitzgerald PC Almeida MD Castro-Alves LS Ahmad S . The prevalence of burnout and depression and their association with adherence to safety and practice standards. Anesth Analgesia. (2013) 117:182–93. doi: 10.1213/ANE.0b013e3182917da9 23687232

[49] Mata DA Ramos MA Bansal N Khan R Guille C Di Angelantonio E . Prevalence of depression and depressive symptoms among resident physicians. JAMA. (2015) 314:2373. doi: 10.1001/jama.2015.15845 26647259 PMC4866499

[50] Pereira-Lima K Loureiro SR . Burnout, anxiety, depression, and social skills in medical residents. Psychology Health Med. (2015) 20:353–62. doi: 10.1080/13548506.2014.936889 25030412

[51] Pereira-Lima K Loureiro SR Crippa JA . Mental health in medical residents: relationship with personal, work-related, and sociodemographic variables. Rev Bras Psiquiatria. (2016) 38:318–24. doi: 10.1590/1516-4446-2015-1882 PMC7111348 27192216

[52] Pereira-Lima K Gupta RR Guille C Sen S . Residency program factors associated with depressive symptoms in internal medicine interns. Acad Med. (2019) 94:869–75. doi: 10.1097/ACM.0000000000002567 PMC6538448 30570500

[53] Shapiro SL Shapiro DE Schwartz GER . Stress management in medical education. Acad Med. (2000) 75:748–59. doi: 10.1097/00001888-200007000-00023 10926029

[54] West CP . Association of resident fatigue and distress with perceived medical errors. JAMA. (2009) 302:1294. doi: 10.1001/jama.2009.1389 19773564

[55] Sen S Kranzler HR Krystal JH Speller H Chan G Gelernter J . A prospective cohort study investigating factors associated with depression during medical internship. Arch Gen Psychiatry. (2010) 67:557. doi: 10.1001/archgenpsychiatry.2010.41 20368500 PMC4036806

[56] Brooks SK Webster RK Smith LE Woodland L Wessely S Greenberg N . The psychological impact of quarantine and how to reduce it: rapid review of the evidence. Lancet. (2020) 395:912–20. doi: 10.1016/S0140-6736(20)30460-8 PMC7158942 32112714

[57] Hauck Filho N MaChado W de L Teixeira MAP Bandeira DR . Evidências de validade de marcadores reduzidos para a avaliação da personalidade no modelo dos cinco grandes fatores. Psicologia: Teoria e Pesquisa. (2012) 28:417–23. doi: 10.1590/S0102-37722012000400007

[58] Santos IS Tavares BF Munhoz TN Almeida LSP da NTB Tams BD . Sensibilidade e especificidade do Patient Health Questionnaire-9 (PHQ-9) entre adultos da população geral. Cadernos Saúde Pública. (2013) 29:1533–43. doi: 10.1590/0102-311X00144612 24005919

[59] Moretti-Pires RO Corradi-Webster CM . Adaptação e validação do Alcohol Use Disorder Identification Test (AUDIT) para população ribeirinha do interior da Amazônia, Brasil. Cadernos Saúde Pública. (2011) 27:497–509. doi: 10.1590/S0102-311X2011000300010 21519700

[60] MaChado W . Escala de bem-estar psicológico: adaptação para o português brasileiro e evidências de validade. (2010). Available at: https://lume.ufrgs.br/handle/10183/29716.

[61] Gygax G Arneson D . Dungeons & Dragons. Lake Geneva, Wisconsin: Tactical Studies Rules (1974).

